# From practice employee to (co-)owner: young GPs predict their future careers: a cross-sectional survey

**DOI:** 10.1186/s12875-017-0591-7

**Published:** 2017-02-02

**Authors:** Luzia Birgit Gisler, Marius Bachofner, Cora Nina Moser-Bucher, Nathalie Scherz, Sven Streit

**Affiliations:** 1 Institute of Primary Health Care (BIHAM), University of Bern, Gesellschaftsstrasse 49, 3012 Bern, Switzerland; 2 Family practice, Nottwil, Switzerland; 3 Center for Primary Care Medicine, University of Basel, Basel, Switzerland; 4 Institute of Primary Care, University Hospital Zurich, University of Zurich, Zurich, Switzerland; 5 Arud Centres for Addiction Medicine, Zurich, Switzerland

**Keywords:** General practice, Practice, Future generation

## Abstract

**Background:**

In Switzerland, the mean age of GPs in 1993 was 46. In 2015, it had increased to 55, and GPs over 65 made up 15% of the workforce of the about 6000 GPs. As older, self-employed GPs retire, young doctors will be needed to fill their positions and eventually take over their practices. We set out to determine what kind of employment young GPs wanted, if they thought their preference would change over time, and the working conditions and factors most important in their choice of practice.

**Methods:**

We administered a cross-sectional online survey to members of the Swiss Young General Practitioners Association (*n* = 443). Our survey relied on closed questions, ratings of attractiveness of fictional job ads, and an open question to capture participants’ characteristics, and their preferred type of practice and working conditions.

**Results:**

We received 270 (61%) replies. Most were women (71%) and wanted to work in the suburbs or countryside in small GP-owned group practices, with up to five colleagues. Most intended to work part-time: mean desired workload was 78% for men and 66% for women. Positive working climate was a major factor in choosing a GP practice. Most participants projected a career arc from employment to ownership or co-ownership of a practice within five years; only 7–9% preferred to remain employees.

**Conclusions:**

Young and future GPs in Switzerland want to work part-time in small, GP-owned group practices. Practices should offer them employment opportunities with a path to (co-)ownership.

**Electronic supplementary material:**

The online version of this article (doi:10.1186/s12875-017-0591-7) contains supplementary material, which is available to authorized users.

## Background

In Switzerland, the mean age of General Practitioners (GPs) in 1993 was 46 [1]. In 2015, mean age of the about 6000 GPs increased to 55, and GPs over 65 made up 15% of the workforce [2]. Today, Swiss GPs are 74% male, 94% are self-employed, and 41% work in single practices (32% in group, and 27% in double practices) [2]. Switzerland is short about 2000 GPs [2]. As GPs retire over the next ten years, the shortage will increase to 4000, and the country will need young doctors to fill their positions [2]. The wave of retirements will open opportunities for young GPs to take over these practices, whether they are ready to do so or not. In 2011, our study of young and future GPs found 41% wanted to be employees rather than practice owners [3]. As a result, some journalists declared that young physicians were not brave enough to venture out on their own [4]. Some older GPs stated that young GPs were unwilling to take risks [5, 6]. Some stakeholders blamed the increasing shortage of GPs on the ‘feminisation’ of the medical profession, arguing women preferred part-time work or were more likely to drop out of the work force [5–11].

We suspected our original study did not tell the whole story, and decided to refine and improve our questionnaire to determine if young GPs wanted to remain employees, or if they thought their preference would change over time. We also set out to identify the most important factors in their choice of practice.

## Methods

### Design

Cross-sectional study using an online survey (Additional file 1).

### Study population

Switzerland has no national register of GP trainees. We used a list provided by the Swiss Young General Practitioners Association (JHaS), the association of young and future GPs. This list was the most complete available. All 456 JHaS members were medical students (3^rd^ year on), residents, or doctors who became GPs within the last five years. Most members were women (69%). Swiss GP trainees are mostly trained in hospitals, though training in GP offices has become more common in the last ten years. Since 2011, residents have been required to spend at least six months training in a GP office, and can spend up to two of their five years there. Medical students were included as they spend several weeks in general practice as part of the curriculum and thus have sufficient experience to comment.

### Processes and outcomes

In Part A, we reproduced four questions about personal characteristics from our 2011 survey [3] and added seven new questions about language, current work, relationships and children. Participants anonymously gave us their residential postal code. In Part B (desired form of practice and working conditions), three of four questions were unchanged: desired workload, practice type, and practice location. We did not define the terms “town”, “suburb” and “countryside”. We rephrased one question to distinguish young GPs’ desires about their first job from their long-term desires. We added six questions to Part B to capture the size of group practice, billing methods, and house calls they desired.

We added two sections. Part C included eight job ads from fictional GP offices so we could identify factors that made practices more attractive. Attractiveness of the job ads was rated on a 10-point Likert scale (1 = very unattractive; 10 = very attractive). Job ads differed in three features: 1) GP-owned or not; 2) small or large office; and, 3) on-site dispensation of medications. Part D was an open question: participants named their three most important factors in choice of practice. The survey was in German or French, spoken by 86% of the Swiss population [12].

The survey was hosted on SurveyMonkey® (www.surveymonkey.com, Palo Alto, CA, USA). A small JHaS committee pilot-tested the survey. In February 2016, we emailed invitations to all JHaS members with valid email addresses (97.3%). We encouraged response in four ways: 1) the study was announced in the JHaS email newsletter; 2) the JHaS president emailed members to encourage participation; 3) we attracted participants by the opportunity to enter a lottery; and, 4) we sent three reminders to non-responders.

### Statistical analysis

We compared baseline characteristics of young GPs (Part A), and the type of practice and working conditions they preferred (Part B). We performed a Chi-square test for categorical data, and t-test or Wilcoxon-Ranksum test for continuous data.

To determine the attractiveness of the eight job ads (Part C), we divided them into groups based on their characteristics, and then averaged their ratings. (For example, we compared the attractiveness of GP-owned and non-GP-owned practices by calculating means of the ratings of the four ads from GP-owned practices, and then calculating means of the ratings of ads from non-GP-owned practices.) We then calculated proportions; for example, we determined the percentage of young GPs who preferred GP-owned practices over non-GP-owned practices. All participants who rated the four ads for GP-owned practices the highest were categorized as preferring GP-owned practices. We calculated proportions and 95% confidence intervals (CI) separately in the univariate model.

In the multivariate model, we used a hierarchical stepwise backward regression to account for possible confounding of ownership, size, and on-site dispensation of medications. Because gender and age are important cofactors, we kept them in the multivariate model. Our comparison of the models was based on the Akaike information criterion (AIC). We decided which cofactor to eliminate, based on the model with the lowest AIC. We used forest plots to calculate random effects for size of group practice, on-site dispensation, and ownership of the GP office. They show odds ratios and 95% CI of the selected cofactors.

Part D took a qualitative approach. We coded and categorized answers to our open question about the three most important factors in a young GP’s choice of practice. We then iteratively sorted answers into categories. We counted their frequency and stratified results by age (young/old), gender, educational level (medical student, resident, GP), and whether they had children.

To determine differences and similarities between the 2011 and 2016 surveys, we compared answers to the same questions. Then we performed Chi2-tests to compare categorical data, and t-tests to compare continuous data. We considered a *p*-value of 0.05 to be statistically significant. We analysed all data with STATA release 14.2 (Stata Corp, College Station, TX, USA).

## Results

### Baseline characteristics (Part A)

Table 1 shows participants’ baseline characteristics. We contacted 443 members of JHaS; 270 (61%) responded to the online survey. Most were women (71%). Overall mean age was 32.9 (SD 4.7). Most respondents were married or had a partner. Every third respondent had children and, regardless of respondent’s gender, most children needed daycare. Of all respondents, 8% were medical students, 40% were young GPs and 52% were residents. Current workload ranged from 60 to 100% (median: 100%). The median was significantly higher for men (100% vs. 80% for women; *p* < 0.001).

**Table 1:** Participants’ characteristics

Characteristics	Overall *n* = 262	Female *n* = 186 (71%)	Male *n* = 76 (29%)	*p*-value
Age, mean (SD)	32.9 (4.7)	32.3 (4.7)	34.3 (4.5)	0.003
Language, no. (% per column)				0.52
German	242 (91)	171 (92)	68 (89)	
French	23 (9)	15 (8)	8 (11)	
Relationship status, no. (% per column)^a^				0.30
Single	49 (19)	37 (20)	12 (16)	
With partner	101 (39)	66 (36)	35 (46)	
Married	110 (42)	81 (44)	29 (38)	
Children, no. (% per column)				0.39
Yes	93 (36)	63 (34)	30 (39)	
No	169 (65)	123 (66)	46 (61)	
Number of children, no. (% per column)				0.15
1	33 (35)	25 (40)	8 (27)	
2	42 (45)	28 (44)	14 (47)	
3	16 (17)	10 (16)	6 (20)	
> 3	2 (2)	0 (0)	2 (7)	
Children in daycare, no. (% per column)^b^				0.09
No	16 (17)	7 (11)	9 (30)	
1 day per week	14 (15)	8 (13)	6 (20)	
2 days per week	35 (38)	27 (43)	8 (27)	
3 days per week	20 (22)	14 (22)	6 (20)	
4 days per week	6 (6)	6 (10)	0 (0)	
5 days per week	2 (2)	1 (2)	1 (3)	
Educational level, no. (% per column)				0.25
Medical student	22 (8)	19 (10)	3 (4)	
Resident	135 (52)	94 (51)	41 (54)	
General practitioner	105 (40)	73 (39)	32 (42)	
Workplace, no. (% per column)^c^				0.18
Medical school	21 (9)	18 (11)	3 (4)	
Hospital	115 (48)	75 (46)	40 (53)	
GP office	104 (43)	71 (43)	33 (43)	
Workload in %, median (IQR)^d^	100 (60–100)	80 (50–100)	100 (95–100)	<0.001

^a^2 participants were divorced

^b^Including care by relatives

^c^Other e.g., on vacation, between jobs, maternity leave, in school: 22 participants

^d^100% equals 50 h/week

### Desired form of practice and working conditions (Part B)

Table 2 shows what participants wanted in a future GP office. Women and men answered similarly for practice type (86% group; 11% double; 2% single practice), number of colleagues (48% chose 4–5; 45% chose 2–3; 6% chose >5), practice location (43% countryside; 40% suburb; 17% town), and also gave similar estimates of their start date as a GP (28% already working; 47% working within 5 years; 23% later). Most respondents said “yes” or “probably yes” (76%) when asked if they would make house calls outside regular on-call hours. Men and women wanted to work part-time, but men preferred a higher workload than women (*p* < 0.001). A large majority of both genders (89%) wanted to be employees in their first GP job (93% of women; 80% of men; *p* = 0.001). Men envisioned being employed for less time (<2 years; 64%) than women (2–5 years; 50%; *p* = 0.005). Only 7 to 9% of all young GPs wanted to be employed throughout their careers. Most wanted to become owners (72%) or co-owners (32%) of a practice (participants could give more than one answer to this question, so percentages total more than 100%).

**Table 2:** Desired form of practice and working conditions

Practice and job characteristics	Overall *n* = 254	Female *n* = 180 (71%)	Male *n* = 74 (29%)	*p*-value
Practice type, no. (% per column)				0.96
Single practice	6 (2)	4 (2)	2 (3)	
Double practice	29 (11)	21 (12)	8 (11)	
Group practice	219 (86)	155 (86)	64 (86)	
If group practice: number of colleagues, no. (% per column)				0.17
2–3	98 (45)	74 (48)	24 (38)	
4–5	106 (48)	74 (48)	32 (50)	
6–10	12 (5)	6 (4)	6 (9)	
10–15	2 (1)	1 (1)	1 (2)	
> 15	1 (0)	0 (0)	1 (2)	
Practice location, no. (% per column)				0.17
Town	43 (17)	29 (16)	14 (19)	
Suburb	102 (40)	67 (37)	35 (47)	
Countryside	109 (43)	84 (47)	25 (34)	
When do you want to start working as a GP? no. (% per column)				0.60
Already working in a practice	72 (28)	48 (27)	24 (32)	
In < 1 year	16 (6)	10 (6)	6 (8)	
In 1–2 years	37 (15)	27 (15)	10 (14)	
In 3–5 years	65 (26)	45 (25)	20 (27)	
In 6–10 years	51 (20)	39 (22)	12 (16)	
In > 10 years	7 (3)	5 (3)	2 (3)	
I don’t know	6 (2)	6 (3)	0 (0)	
House calls, no. (% per column)				0.28
Yes	95 (37)	65 (36)	30 (41)	
Probably yes	98 (39)	72 (40)	26 (35)	
Probably not	37 (15)	28 (16)	9 (12)	
No	6 (2)	2 (1)	4 (5)	
I don’t know	18 (7)	13 (7)	5 (7)	
Desired workload in %, mean (SD)^a^	70 (15.8)	66 (15.1)	78 (14.2)	<0.001
Remuneration, no. (% per column)				<0.001
Fixed salary based on workload	45 (18)	41 (23)	4 (5)	
Billing for my own time	81 (32)	44 (24)	37 (50)	
Part fixed, part share of the revenue	122 (48)	93 (52)	29 (39)	
Only share of the revenue	6 (2)	2 (1)	4 (5)	
Employment of first job as GP, no. (% per column)^b^				0.001
Employed	227 (89)	168 (93)	59 (80)	
Self-employed	27 (11)	12 (7)	15 (20)	
If employed: time frame of being employed, no. (% per column)				0.005
< 2 years	106 (47)	68 (40)	38 (64)	
2–5 years	98 (43)	84 (50)	14 (24)	
6–10 years	8 (4)	4 (2)	4 (7)	
10–15 years	0 (0)	0 (0)	0 (0)	
My whole career	15 (7)	12 (7)	3 (5)	
Long-term desires, no. (%)				
Be employed	25 (9)	22 (12)	3 (4)	0.049
Be employed co-owner	84 (32)	69 (37)	15 (20)	0.006
Own the practice	192 (72)	130 (70)	62 (82)	0.052
Medical director of a large group practice	29 (11)	12 (6)	17 (22)	<0.001
Chief administrater of a group practice	19 (7)	3 (2)	16 (21)	<0.001

^a^100% equals 50 h / week

^b^Desired if not yet in a practice, resp. how it was if already in a practice

### Attractiveness of eight fictional job advertisements from GP offices (Part C)

**Fig. 1:**
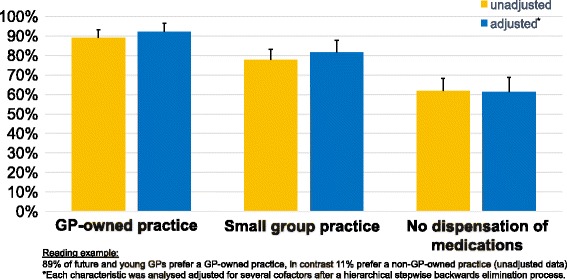
Preferences of future and young GPs

The mean score of the Likert scale ratings (10 = very attractive) for all ads was 6.1 (SD 1.4; data not shown). After we separated the ads into two groups of four (based on office size), the univariate model showed that 78% of respondents (95% CI 72–83%) preferred a small GP office (Fig. 1). Most (62%; 95% CI 55–68%) preferred offices that did not dispense medications on-site. A large majority (89%; 95% CI 84–92%) preferred working in a GP-owned practice. We adjusted the multivariate model for several cofactors, with similar results.

**Fig. 2:**
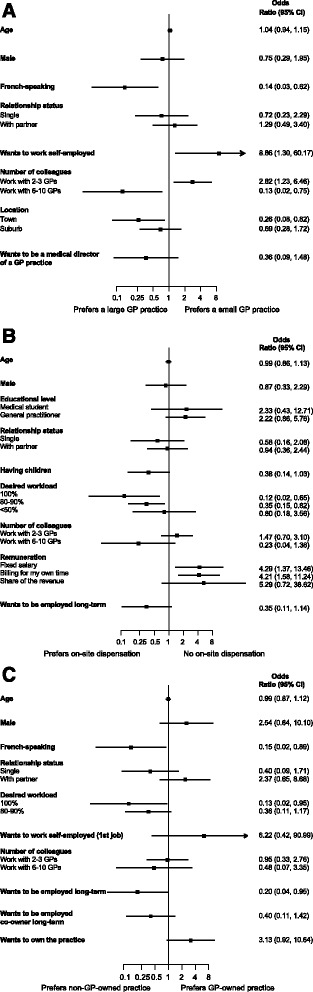
Associations between selected characteristics and probability to prefer a small GP practice (Panel **a**), no on-site dispensation of medications (Panel **b**), and GP-owned practice (Panel **c**). Shows odds ratios with corresponding 95% confidence intervals from multivariate logistic regression models after hierarchical stepwise backward elimination process of covariates

Figure 2 contains forest plots for our three features. Preferring a small practice was independently associated with wanting to be self-employed (OR 8.86, 95% CI 1.30–60.17) and wanting to work in an office of 3 to 4 physicians (OR 2.82, 95% CI 1.23–6.46) (Fig. 2, Panel a). Those who preferred working in a town (OR 0.26, 95% CI 0.08–0.82), and French-speaking participants (OR 0.14, 95% CI 0.03–0.62), preferred large practices. Participants who preferred practices that did not dispense medications preferred a fixed salary (OR 4.29, 95%CI 1.37–13.46) or wanted to bill for their own time (OR 4.21, 95% CI 1.58–11.24) (Fig. 2, Panel b). Those who wished to work 80 to 90% (OR 0.35, 95% CI 0.15–0.82) or 100% (OR 0.12, 95% CI 0.02–0.65) preferred practices that dispensed medications (Fig. 2, Panel c). French-speaking respondents (OR 0.15, 95% CI 0.02–0.89), those who wanted to work 100% (OR 0.13, 95% CI 0.02–0.95), and those who wanted to remain employees in the long-term (OR 0.20, 95% 0.04–0.95) preferred to work in non-GP-owned practices.

### Three most important factors in choice of a GP practice (Part D)

When asked to write in the top three factors in their choice of future GP practice, participants emphasised seven factors (Table 3). Working climate (e.g., pleasant colleagues, working well together, good team work) were among the three answers of 72% of all respondents. Other important factors included location (52%), workload (34%), infrastructure (22%), group practice (14%), self-employment (14%), and spectrum of services (11%). These factors accounted for 74% of all answers. The answers of subgroups matched on everything but self-employment: older participants mentioned self-employment more often than younger participants.

**Table 3:** Three most important factors in choice of a GP practice

Within top three answers	Category	Mentioned most frequently
72%	Working climate	Pleasant colleagues, working well together, good team work
52%	Location	Near home, rural
34%	Workload	Part-time, flexible or well-regulated working hours
22%	Infrastructure	Rooms, equipment, diagnostics, IT
14%	Group practice	Group or common practice
14%	Self-employment^a^	Self-employed, independent
11%	Spectrum of services	Wide-ranging, interdisciplinary, complementary medicine

^a^More older participants (20%) chose self-employment than younger participants (7%) (*p* = 0.005)

### Comparing the 2016 survey to the 2011 survey

Participants answered the same questions on the 2011 and 2016 survey similarly (Table 4). But, in 2016, when we divided our question about prospective employment (desires about their first job vs. their long-term goals), this markedly changed our results. When we asked the question without a time frame in 2011, 41% preferred employee status. But in 2016, 89% of young GPs preferred to take a *first* job as an employee. The number who wanted to be employees in the long-term dropped precipitously (7–9%); the large majority envisioned themselves owning or co-owning a practice within five years.

**Table 4:** Comparing the 2016 survey to a smaller 2011 survey [3]

Characteristics	2016 *n* = 262	2011 *n* = 104	*p*-value
Age, mean (SD)	32.9 (4.7)	31.8 (5.7)	0.06
Female, no. (%)	186 (71)	71 (68)	0.57
Educational level, no. (% per column)			0.01
Medical student	22 (8)	20 (19)	
Resident	135 (52)	50 (48)	
General practitioner	105 (40)	34 (33)	
Practice type, no. (% per column)			0.10
Single practice	6 (2)	6 (6)	
Double practice	29 (11)	17 (16)	
Group practice	219 (86)	81 (78)	
Practice location, no. (% per column)			0.29
Town	43 (17)	18 (17)	
Suburb	102 (40)	33 (32)	
Countryside	109 (43)	53 (51)	
Employment of first job as GP^a^, no. (% per column)			<0.001
Employed	227 (89)	43 (41)	
Self-employed	27 (11)	61 (59)	
Desired workload in %, mean (SD)	70 (15.8)	72 (16.7)	0.28

^a^This is from the 2016 survey. The question was phrased slightly differently in 2011

## Discussion

### Summary

Most respondents (71%) were women; most wanted to work in the suburbs (40%) or countryside (43%). Young GPs wanted to work in small GP-owned group practices, with up to five colleagues. Most intended to work part-time (mean average desired workload was 78% for men and 66% for women); 72% felt that positive working climate was a major factor in choosing a GP practice; and 76% were willing to do house calls outside regular on-call hours, a typical but not mandatory task of Swiss GPs. Dispensing medications made a practice more attractive for over a third of respondents (38%). Most young doctors projected a career arc from employment (89%) to ownership or co-ownership of a practice. Only 7 to 9% preferred to remain employees.

### Strengths and limitations

Our high response rate (61%) was a strength, as were the different approaches we took: fictional job advertisements, closed questions, and an open question. Combining direct and indirect questions made the career desires of young GPs clearer. We changed only one question from our 2011 survey, so comparing older data to new data, and comparing snapshots in time was straightforward. We added new questions to clarify points the 2011 study had obscured. We were limited by the hypothetical nature of the survey, but the results of our 2011 survey suggest that physicians’ predictions may be accurate. For example, the number of group practices is increasing, and more GPs work part-time [13]. Our 2016 results may similarly describe a trend in primary care in Switzerland. We were limited by the fact Switzerland has no national register, and basing the survey on JHaS respondents may have resulted in some selection bias (JHaS members might be more active than their non-member peers). Our results cannot be generalized to the Italian-speaking region of Switzerland.

### Comparison with existing literature

Both our 2011 and 2016 results showed young Swiss GPs clearly preferred group practices, especially small group practices. International studies demonstrated a trend towards replacing single practices with group practices [2, 14–16]. We found no other studies that tracked associations between desire for self-employment and preference for smaller GP offices. We think we are the first to associate a preference for town practices (over countryside/suburb), and French speaking status, with working in larger GP offices.

Switzerland, like many other countries, has a shortage of GPs, particularly in some rural areas, where the number of GPs per 1000 inhabitants is less than a fifth of the number per 1000 in urban areas [17, 18]. In some other countries, young doctors disliked rural practices, but 43% of our respondents wanted to work in the countryside [19–22]. This should be good news, but we do not know which cantons or communities they prefer, and cannot be sure that their eventual distribution will match our needs.

In Switzerland, women make up more than half of medical school graduates [23]. Other studies showed more women entering primary care than men [24, 25]. Of our respondents, 71% were women. This explains only part of the increase in desire for part-time work. We found both genders preferred part-time work (mean desired workload: men 78%, women 66%). This, and the slight decrease in GP workload over time (51.2 h/week in 2005; 47.9 h/week in 2015), might indicate a generational shift [2]. For example, a 2015 study of Swiss medical residents of all specialties found that, independent of gender, almost half (47%) wanted to reduce their workload [26]. Reasons for this wish were mainly the better compatibility of family and occupation (66%). Other reasons were longer recovery phases (18%) and more leisure time (15%) [26].

## Implications for research and practice

If Switzerland established a national register of GP trainees, we could track and more accurately project the career choices and arc of young GPs, and increase the external validity of studies like ours. Since many young GPs have or are planning to have children, we also need to study the intersections between career and family life.

One of the best ways to attract young GPs to Swiss practices is to offer them the option of part-time work. Practices and teaching hospitals that host GP residents should offer young physicians more opportunities for part-time work. Retiring GPs and recruiters for practices should think about converting or merging private practices, and should offer opportunities for young physicians to transition from employee to (co-)owner. A large minority of GPs, especially those who want to work 80 to 100%, also want to dispense medications, so this should remain an option. Practices should take regional preferences into account when they create jobs and offer ownership opportunities.

Stakeholders who claim that young doctors prefer employment to owning a practice are telling only part of the truth. Since the projected career span of a GP is around 30 years (mean age at finishing residency was 36.6 [23]), young GPs intend to spend a relatively short time as employees and most of their career as owners or co-owners. This initial desire for employment must be acknowledged and met, but it should also be understood as a career stage, rather than a permanent condition. While young Swiss GPs may not be ready to take over practices as quickly as older physicians retire, and may prefer small group practices to solo practices, this does not mean they lack initiative or the spirit of entrepreneurship. Our study demonstrates that the large majority of young GPs project a career arc that begins as an employee, but transitions to self-employment within five years.

## Conclusions

Young and future GPs in Switzerland want to work in small, GP-owned group practices, with up to five colleagues. Most aim for practices in the suburbs or countryside and both female and male GPs intend to work part-time. The majority feels that positive working climate is a major factor in choosing a GP practice. The possibility to dispense medications makes a practice more attractive for over a third of the respondents. Most young doctors project a career arc from employment to ownership or co-ownership of a practice within five years. Only a small minority prefers to remain employees. This initial desire for employment must be acknowledged and met, but it should also be understood as a career stage, rather than a permanent condition.

## Additional file

Additional file 1: Online survey used in this study. (DOCX 21 kb)

## Abbreviations

AIC: Akaike information criterion
CI: Confidence interval
GP: General Practitioner
HFG: Humanforschungsgesetz (Swiss law on human research)
IQR: Interquartile range
JHaS: Junge Hausärzte Schweiz (Swiss Young General Practitioners Association)
OR: Odds ratio
SD: Standard deviation

## References

[1] Cohidon C, Cornuz J, Senn N (2016). Die Entwicklung der Hausarztmedizin in der Schweiz. Prim Hosp Care.

[2] Zeller A. Work Force Studie 2015: «Den Puls der Schweizer Hausärzte wissenschaftlich gefühlt» Retrieved from http://www.synapse-online.ch/uploads/media/22_Work_Force_Studie_2015_Den_Puls_der_Schweizer_Hausaerzte_wissenschaftlich_gefuehlt.pdf. Accessed 26 Jan 2017.

[3] Streit S (2011). Moderne Praxisformen. Prim Care.

[4] Von Borstel S. Der Hausarzt verkommt zum Auslaufmodell. Die Welt; 2014. Retrieved from https://www.welt.de/debatte/kommentare/article131910670/Der-Hausarzt-verkommt-zum-Auslaufmodell.html. Accessed 6 Jan 2017.

[5] Paone A. Anschubhilfe für junge Hausärzte. Basler Zeitung; 2015. Retrieved from http://bazonline.ch/basel/gemeinden/Anschubhilfe-fuer-junge-Hausaerzte/story/28582438. Accessed 6 Jan 2017.

[6] Furrer P. Junge Ärzte wollen heute lieber angestellt als selbstständig sein. Oltner Tagblatt; 2012. Retrieved from http://www.oltnertagblatt.ch/solothurn/grenchen/junge-aerzte-wollen-heute-lieber-angestellt-als-selbststaendig-sein-119102499. Accessed 6 Jan 2017.

[7] Altermatt S. Die Hausärzte stecken mitten in einer Revolution. Solothurner Zeitung; 2014. Retrieved from http://www.solothurnerzeitung.ch/solothurn/kanton-solothurn/die-hausaerzte-stecken-mitten-in-einer-revolution-127937049. Accessed 15 Dec 2016.

[8] Maurer L (2014). Löst die Gemeinschaftspraxis die klassische Einzelpraxis ab?. Schweizerische Ärztezeitung.

[9] Loretan L, van der Heiden N, Kraft E (2016). Jeder zehnte Arzt steigt aus. VSAO J.

[10] Bracher K. Jeder fünfte Arzt wechselt den Beruf. NZZ am Sonntag; 2015. Retrieved from http://www.nzz.ch/nzzas/nzz-am-sonntag/jeder-fuenfte-arzt-wechselt-den-beruf-ld.1915. Accessed 6 Jan 2017.

[11] Reichmuth A. Fehlende Spezialisten. Die Weltwoche; 2011. Retrieved from http://www.weltwoche.ch/ausgaben/2011_32/artikel/fehlende-spezialisten-die-weltwoche-ausgabe-322011.html. Accessed 19 Dec 2016.

[12] Bundesamt für Statistik. Als Hauptsprachen genannte Sprachen, 2014. 2016. Retrieved from http://www.bfs.admin.ch/bfs/portal/de/index/themen/01/05/blank/key/sprachen.html. Accessed 6 Jan 2017.

[13] Forster C. Boomende Gruppenpraxen. Neue Zürcher Zeitung; 2016. Retrieved from http://www.nzz.ch/schweiz/aktuelle-themen/managed-care-ld.115043. Accessed 6 Jan 2017.

[14] Strandberg-Larsen M, Nielsen MB, Vallgårda S (2007). Denmark: Health system review. Health Syst Transit.

[15] Baudier F, Bourgueil Y, Evrard I (2010). Group practice dynamics among private general practitioners from 1998 to 2009. Questions d’économie de la Santé.

[16] Hsiao CJ, Cherry DK, Beatty PC, Rechtsteiner EA (2010). National ambulatory medical care survey: 2007 summary. Natl Health Stat Rep.

[17] Hostettler S, Kraft E (2016). Zuwanderung grundlegend für Versorgungssystem. Schweizerische Ärztezeitung.

[18] Ono T, Schoenstein M, Buchan J (2014). Geographic imbalances in doctor supply and policy responses. OECD Health Working Papers.

[19] Jacob R, Kopp J, Schultz S. Berufsmonitoring Medizinstudenten 2014. 2015. Retrieved from http://www.kbv.de/html/5724.php. Accessed 6 Jan 2017.

[20] Lu DJ, Hakes J, Bai M (2008). Rural intentions: factors affecting the career choices of family medicine graduates. Can Fam Physician.

[21] Rabinowitz HK, Diamond JJ, Markham FW, Santana AJ (2012). The relationship between entering medical students’ backgrounds and career plans and their rural practice outcomes three decades later. Acad Med.

[22] Scott A, Witt J, Humphreys J (2013). Getting doctors into the bush: general practitioners’ preferences for rural location. Soc Sci Med.

[23] FMH Swiss Medical Association. Poster FMH-Ärztestatistik 2015. Retrieved from http://www.fmh.ch/files/pdf17/FMH-Aerztestatistik_2015_Poster_D.pdf. Accessed 6 Jan 2017.

[24] Vanasse A, Orzanco MG, Courteau J, Scott S (2011). Attractiveness of family medicine for medical students: influence of research and debt. Can Fam Physician.

[25] Gill H, McLeod S, Duerksen K, Szafran O (2012). Factors influencing medical students’ choice of family medicine: effects of rural versus urban background. Can Fam Physician.

[26] Hess B (2016). Ein Drittel will Teilzeit arbeiten. VSAO J.

