# Supplementary material for: From practice employee to (co-)owner: young GPs predict their future careers: a cross-sectional survey
Source: BMC Fam Pract. 2017 Feb 2;18:12. doi: 10.1186/s12875-017-0591-7 (PMC5289023; doi:10.1186/s12875-017-0591-7)
Supplement: Additional file 1: — Online survey used in this study. (DOCX 21 kb) [file 12875_2017_591_MOESM1_ESM.docx]

**Additional file 1.** Online survey

Part A

- Choose your language o German o French

- Gender o Female o Male

- Birth year (e.g. 1980) _______

- Residential postal code (e.g. 3010) _______

- Current educational level o Medical student o Resident o GP

- Current workplace o Medical school o Hospital o GP office

o Other: ________

- Current workload in % (100% equals 50 hrs / week) o 0% o 10% o 20% o 30% o 40% o 50% o 60% o 70% o 80% o 90% o 100%

- Relationship status o Single o With partner

o Married o Other: _______

- Do you have children? o Yes o No

- If you have children:

- How many children do you have? o 1 o 2 o 3 o >3

- How many days per week are your children in daycare (including care by relatives, please round off

to whole numbers) o 1 o 2 o 3 o 4 o 5

Part B

- Which type of practice do you most prefer? o Single practice o Double practice

o Group practice o I won’t work as a GP

- If you answered “group practice”:

- How many physicians do you want to work with? o 2-3 o 4-5 o 6-10 o 10-15 o >15

- When do you want to start working in a GP practice? o I already work in a practice

o in <1 year o in 1-2 years

o in 3-5 years o in 6-10 years

o in >10 years o I don’t know

- Where do you want to work? o Town o Suburb o Countryside

- Your desired workload in % o 10% o 20% o 30% o 40% o 50%

(100% equals 50 hrs/week) o 60% o 70% o 80% o 90% o 100%

- What do you prefer? o Fixed salary based on workload

o Billing for my own time

o Part fixed, part share of the revenue

o Only share of the revenue

- I will make house calls (outside regular on-call hours) o Yes o Probably yes o I don’t know

o Probably not o No

- Which do you prefer for your first job as a GP? If you are already a GP, which was your first job?

o Employed o Self-employed

- Based on your answer to the previous question, how long would you to remain self-employed or employed before changing your status? o <2 years o 2-5 years o 6-10 years

o 10-15 years o My whole career

- What are your long-term desires? (Multiple answers possible)

o Be employed o Be employed co-owner

o Own the practice

o Medical director of a large group practice

o Chief administrator of a group practice

o Other: _______

Part C

We will show you 8 consecutive job advertisements from GP offices. They are similar, so please read them carefully. Please rate their attractiveness as if you were looking for such a job.

**After each advertisement you will be asked:**

- How attractive is this job offer? o 1 o 2 o 3 o 4 o 5

(Scale: 1 = very unattractive to 10 = very attractive) o 6 o 7 o 8 o 9 o 10

**Advertisement 1 and 2** (advertisement 2 is without on-site dispensation of medications)

The medical centre Medilife is looking for a

General Practitioner

to complete the physician team

(Full- or part-time possible)

We offer:

- Large interdisciplinary team with a lot of physicians and practice nurses

- On-site dispensation of medications

- Opportunity to become a partner in our GP-owned medical centre

- Broad spectrum of challenging work

Please send your application to:

Jonas Meier, MD

Medical Director of Medilife

**Advertisement 3 and 4** (advertisement 4 is without on-site dispensation of medications)

We are looking for a

General Practitioner

for our small, well established group practice

(Full- or part-time possible)

We offer:

- A well-coordinated team with 5 practice nurses

- On-site dispensation of medications

- Opportunity to help determine the future of our nationwide practice group Swissdoc Joint Stock Corp.

- Broad spectrum of challenging work

Please send your application to:

Jan Baumann MBA

HR Department

Practice group Swissdoc Joint Stock Corp.

**Advertisement 5 and 6** (advertisement 6 is without on-site dispensation of medications)

We are looking for a

General Practitioner

for our small, well established group practice

(Full- or part-time possible)

We offer:

- A well-coordinated team with 5 practice nurses

- On-site dispensation of medications

- Opportunity to become a partner in our GP-owned group practice

- Broad spectrum of challenging work

Please send your application to:

Matthias Brunner, MD

General Practitioner

**Advertisement 7 and 8** (advertisement 8 is without on-site dispensation of medications)

The medical centre Primacare Joint Stock Corp. is looking for a

General Practitioner

to complete the physician team

(Full- or part-time possible)

We offer:

- Large interdisciplinary team with a lot of physicians and practice nurses

- On-site dispensation of medications

- Opportunity to help determine the future of our medical centre and the nationwide represented practice group Primacare Joint Stock Corp.

- Broad spectrum of challenging work

Please send your application to:

Andreas Keller, MBA

HR Department

Practice group Primacare Joint Stock Corp.

Part D

- What are 3 most important factors in your choice of a GP practice?

1. _____________

2. _____________

3. _____________

You did it! Thank you very much for your participation in the survey.

Your information will be evaluated anonymously.
